# Licensing of medical biochemists and specialists in laboratory medicine, Croatian academic educated professionals in health care

**DOI:** 10.3352/jeehp.2016.13.4

**Published:** 2016-01-13

**Authors:** Daria Pašalić, Honović Lorena

**Affiliations:** 1 Department of Medical Chemistry, Biochemistry and Clinical Chemistry, University of Zagreb, School of Medicine, Zagreb, Croatia; 2 Department of Laboratory Diagnostics, General Hospital Pula, Pula, Croatia

## Who are medical biochemists?

Croatian health system recognizes the master of medical biochemistry (clinical chemistry) as one of four academically educated professionals in health care including medical doctors, dental medicine doctors, master of pharmacy and master of medical biochemistry/graduate engineers of medical biochemistry. This is determined by a variety of legislative rules. In relation to the medical biochemistry and laboratory medicine it is important to emphasize the ‘Law on medical biochemical activity’ from 2003 [1]. According to that law health professional in medical biochemistry are graduated engineer of medical biochemistry.

Development of medical biochemistry as a profession dates back to the early 20th century and it is linked to the names of professors Ibrahim Ruzdic and Marijana Fisher-Herman [2]. Professor Ruzdic was the first one who established medical-biochemical laboratory in Croatia in 1937. Altogether with his co-workers, doctors, and professors of different laboratory disciplines of laboratory, he organized a one-year training of the academically educated pharmacists, medical doctors, and chemists for the management of the medical laboratories. Professor Fisher-Herman in 1946 established department of clinical chemistry at faculty of pharmacy, University of Zagreb and in 1962 an independent study of medical biochemistry at the same faculty. Specialized and scientific postgraduate study of medical biochemistry dates from 1961. Thanks to a number of highly qualified experts in the field of laboratory medicine, medical biochemists are since 1973 recognized as a health professionals. Then they gained the right to take the professional exam which is eligible for the specialist postgraduate training. In1994 it was established the Croatian Chamber of Medical Biochemists (CCMB) which is legally responsible for the regulation of the profession in Croatia [1].

Since 2007 according the ‘Law on academic and professional titles and academic degrees’ [3] medical biochemists that have completed an integrated university study of medical biochemistry acquires the title master of medical biochemistry. This is in agreement with Bologna declaration from 1999 and the Croatian reform of medical biochemistry studies [4]. This reform was based on the criteria accepted in the majority of the European countries for employment in laboratories of medical biochemistry ‘European Syllabus for Post graduate for the training in Clinical Chemistry’ [4,5]. Additionally, there is a large heterogeneity in education and naming of professionals with academic degree who work in laboratory medicine practice, in Europe and worldwide [6]. Therefore, European Federation for Clinical Chemistry and Laboratory Medicine in consultation with its member societies define the name for undergraduate professionals: specialist in laboratory medicine [6]. Therefore, the students who attend integrated academic study at Zagreb University Faculty of Pharmacy and Biochemistry since 2015 will acquire the title master of medical biochemistry and laboratory medicine.

In Croatia, masters of medical biochemistry or up-to date masters of medical biochemistry and laboratory medicine (in further text: medical biochemists) are bearers of medical biochemistry profession and activities in collaboration with laboratory technicians, bachelors of medical laboratory diagnostics and other health professionals. Medical biochemist is a health care worker who deals with the application of biochemistry, hematology, molecular biology, and chemical research in biological samples in order to determine the causes of disease, health maintenance, disease prevention, and therapy monitoring. To do so, he must meet the general requirements for carrying out medical biochemical activities. These are: diploma of pharmacy and biochemistry in the Republic of Croatia for a master’s degree in medical biochemistry or validated foreign diploma of graduation from the appropriate university level study, passed the certification exam, Croatian citizenship, Croatian language, entry in the register of CCMB and license for independent work. Special conditions for carrying out medical biochemical activities are specialization or sub-specialization in medical biochemistry [1,7]. CCMB is the legislative body that is responsible for, inter alia, the register of medical biochemists in the Croatia, authorizations of independent work, and professional supervision of medical biochemists’ work.

## Licensing of medical biochemists in Croatia

Rights and commitments of medical biochemists include professional continuing training and verification of competence in order to obtain the conditions for renewal licenses for independent work. Professional continuing training includes continuous monitoring of the development of medical biochemistry science, and acquires new knowledge, competencies, skills, and attitudes.

Based on general requirements for carrying out medical biochemical activities, medical biochemists may start with professional work after being registered at the CCMB register and after obtaining the authorization for independent work. They are also obligate to renew their licenses every six years. The conditions for renewal of the license are prescribed by the statute of the CCMB [8]. According to the CCMB’s general act there are three ranges of authorizations for the license: the authorization for independent work of a medical biochemist, the authorization for independent work of a medical biochemist-specialist, and the authorization for independent work of a subspecialist. Recently adopted act, ‘Ordinance on continuous training for medical biochemists’ (2015) includes rules relating to the content, terms and process of continuous education and examination and evaluation of professional training of medical biochemists. The same act provides the activities of The CCMB committee for continuous education which presents one of the major factors in postgraduate continuous education of medical biochemists.

## Continuous postgraduate education forms for continuous professional development

The most important forms of continuous postgraduate education (CPE) defined with the Ordinance are: specialization in medical biochemistry and laboratory medicine (MBLM) or specialization in analytical toxicology, and sub-specializations in MBLM or analytical toxicology. The prerequisites for specializations and sub-specializations are attending postgraduate specialist studies of medical biochemistry and laboratory medicine on the Zagreb University Faculty of Pharmacy and Biochemistry [9]. Other forms of CPE include courses of continuous education, organized visit training and meetings in the clinics, clinical hospitals or clinical hospital centers, attending professional and scientific meetings, conferences and symposia, international sabbaticals, workshops in organization or patronage of CCMB, attending of professional meetings organized by Croatian Society of medical Biochemistry and Laboratory Medicine (CSMBLM) or other societies, attending e-seminars organized by CSMBLM or other societies, publishing of professional and scientific papers, publishing of professional books or book chapters, getting a PhD title, development of patent in the field of medical biochemistry and laboratory medicine, and mentorship for PhD or specialist work [9].

Courses of CPE are mostly organized by CCMB and include all fields of medical biochemistry and laboratory medicine or analytical toxicology: medical biochemistry, hematology, coagulation, immunology, immuno-phenotyping, molecular diagnostics, analytical toxicology and pharmacology, quality management, and laboratory organization and management [9]. Courses are divided onto three categories. The 1st category course processes professional problems and theoretical knowledge for enabling the acquisition of skills in a minimum of six and maximum of eight lectures. It includes written materials and written test; the 2nd category course processes expert issues in at least six lectures. It includes written materials, while written test isn’t mandatory; and the 3rd category course processes expert issues in at least 6 lectures. Written material and written exam aren’t mandatory.

## Renewal and revocation of license

The most of the European countries have defined continuing professional development programmers and corresponding crediting systems which is recognized as an optimal service for the patients provided by laboratory medicine specialists [10]. According to the origin of participation or the category of the event, in the Croatia participant can be assigned for appropriate points by CCMB. In a period of six years medical biochemist can choose the categories and origins of continuous education in accordance to the Ordinance on continuous education [9]. He has to attend different forms of CPE. Maximally 50% of CCMB credits can be achieved with only one CPE form with exceptions for terminated specialization in laboratory medicine or analytical toxicology in the six year period.

CCMB Ordinance on the issue, renewal and revocation of licenses for independent work [11] prescribes that it is mandatory to renew the license every six year. Medical biochemist needs to collect 30 points in that period. It is proposed that for continuous professional improvement, medical biochemists have to collect at least 5 points yearly. Organizer of CPE issues the certificates as documents that serve as the evidence of participation. System for crediting is Ordinance on continuing education [8] and it includes all form of continuous education [9]. There are some similarities between licensing of medical biochemists and Croatian medical doctors who are also obligate to renew their licenses every six years. Evaluation of professional development of medical doctors (MD) for the purpose of renewing the medical licenses is prescribed in the Ordinance on continuing medical education [12]. According to the ordinance MD have to collect 120 points in the similar types of CPEs, because the system and gradation of different CPE types is not the same as for medical biochemists.

## Adaptation to the European common training framework

As already mentioned above, there is a large heterogeneity among European countries in definition of the profession laboratory medicine. Academically educated laboratory professionals across Europe may have their basic education in medicine, pharmacy or science. A postgraduate training lasts from four to seven years while together with academic education it ranges from nine to thirteen [7]. Due to that, 4th version of the European Syllabus for Post-Graduate training in Clinical Chemistry defined goals in harmonization process [5] which includes defining of the education period, specialist training, competences, registration and participation in continuous professional development. Continuous postgraduate education and, continuous professional development in Croatian is focused to the adaptation to the European common training framework and regulation of the laboratory medicine profession.
